# Significance of Brighton Criteria in the Early Diagnosis and Management of Guillain-Barré Syndrome

**DOI:** 10.7759/cureus.8318

**Published:** 2020-05-27

**Authors:** Haider Ghazanfar, Rabia Qazi, Ali Ghazanfar, Sania Iftekhar

**Affiliations:** 1 Internal Medicine, Bronxcare Health System, Bronx, USA; 2 Medicine, Women Medical College Abbottabad, Abbottabad, PAK; 3 Cardiology, Federal Medical and Dental College, Islamabad, PAK; 4 Pharmacology, Shifa College of Medicine, Islamabad, PAK

**Keywords:** syndrome, early diagnosis, female, epidemiology, guillain-barré syndrome

## Abstract

Guillain-Barré syndrome (GBS) is a rare but severe autoimmune disease and the usage of the Brighton criteria can be a source of great help in resource-limited settings. It encompasses all ages. Late diagnosis of GBS can have a significant negative impact on the prognosis. The Brighton criteria are used to assist in the diagnosis of GBS and help distinguish between low-risk and high-risk patients. In this article, we have discussed the challenges regarding the diagnosis of the GBS and the possible solutions that can help in the early diagnosis and management of GBS.

## Editorial

Guillain-Barré syndrome (GBS) is a rare autoimmune disease having a potential of life-threatening complications. It is acute in onset and involves inflammatory polyneuropathy. In this disease, immune-mediated damage of the peripheral nervous system occurs with the destruction of the layer of insulation around the nerve axon, the myelin sheath, and rapidly developing muscle weakness.

GBS is a disease that encompasses all ages but is rarely seen in infants. A number of studies have found the incidence of GBS to be 0.16 to 4/100000 per year [1]. These studies included all age groups. GBS is found to be more prevalent in adults who are older than 50 years of age and is more common in males as compared to females [1].

The diagnosis of GBS is usually a result of the clinical evaluation. The development of the Brighton criteria by the Brighton Collaboration has assisted in making the diagnosis of GBS easier for clinicians. The criteria enlist all of the inceptive symptoms inherent to the disease, as well as the diagnostic tools that help in its identification and monitoring [2]. The different levels of Brighton criteria have been demonstrated in Table 1 [3].

**Table 1:** Brighton criteria for GBS +, present; -, absent; +/−, present or absent; CSF, cerebrospinal fluid; GBS, Guillain-Barré syndrome; NCS, nerve conduction study

Diagnostic criteria	Level of diagnostic certainty			
	Level 1	Level 2	Level 3	Level 4
Absence of alternative diagnosis for weakness	+	+	+	+
Diminished or absent deep tendon reflex in weak limbs	+	+	+	+/−
Monophasic course and time between onset and nadir, 12 hours to 28 days	+	+	+	+/−
Bilateral and flaccid weakness of limbs	+	+	+	+/−
CSF cell count < 50 cells/microL	+	+	-	+/−
CSF protein concentration > normal value	+	+/−	-	+/−
NCS findings consistent with one of the subtypes of GBS	+	+/−	-	+/−

The sensitivity of the Brighton criteria correlates with the levels of the criteria. Roodbol et al. deduced in a study that Levels 1, 2, and 3 had sensitivities of 72%, 96%, and 98%, respectively [4]. In a study conducted in India, it was found that Level 3 of the Brighton criteria helped detect 86% of the cases of GBS, whereas Level 2 detected 84% and Level 1 detected 62% of the cases [5].

The Brighton criteria are used as a tool to assist in diagnosing GBS and help distinguish between low-risk and high-risk patients. They aid in the early and prompt diagnosis of the disease. The criteria also help in outlining the course of treatment required by the particular number of patients diagnosed with GBS. In an ill-equipped clinical setup, the Brighton criteria play a prime role in the detection of GBS.

Currently, few physicians, specialists, residents, and medical students have knowledge regarding the Brighton criteria as a means to diagnose GBS. The lack of awareness and application of the Brighton criteria has made it difficult for physicians to identify clinically suspected cases of GBS. This has resulted in increasing rates of misdiagnosis that eventually poses the risk of fatal complications to the patient. This leads to increased mortality and morbidity resulting in increases in the burden of responsibility on the physician and healthcare facility. To prevent the occurrence of such situations, it is essential that all healthcare-associated individuals be brought up to date regarding the new diagnostic criteria for all diseases, and in this case, GBS. Events of missed diagnosis of GBS can be overcome if knowledge about the Brighton criteria is made available to impending doctors from an early stage of their career. Textbooks that are part of the curriculum of a medical school should mention the Brighton criteria as a means to diagnose and manage GBS at an early onset. Any patient diagnosed with GBS, observed on clinical rotations or ward rounds, is an opportunity to provide medical students and residents with the knowledge of the Brighton criteria and how the criteria should be applied on a case-by-case basis.

For physicians and specialists, it is essential that they should be upgraded regarding the latest diagnostic tools and measures used for diagnosing GBS. The importance of the review of the literature regarding GBS cannot be emphasized for every future and practicing medical practitioner. There is a need to increase awareness of Brighton criteria among physicians. This can be done through available modes of communication like conduction of seminars, workshops, case presentations, pamphlets, articles, and oral discussions among groups of doctors at different hospitals across many different geographic locations. The collective efforts of all physicians together, in increasing the awareness of the Brighton criteria, may help to lower the morbidity and mortality associated with GBS and somewhat lessen the burden of this disease.
